# Near-total jejunoileal atresia with exteriorized cecum and gangrenous appendix associated with gastroschisis: a case report

**DOI:** 10.1097/RC9.0000000000000522

**Published:** 2026-05-07

**Authors:** Darrick L. Yeta, Andinet Dessalegn, Mikyas B. Nigusse, Zekarias Leta

**Affiliations:** a Department of General Surgery, MCM Comprehensive Specialized Hospital, Addis Ababa, Ethiopia; b Department of Medicine, Myungsung Medical College, Addis Ababa, Ethiopia

**Keywords:** case report, gastroschisis, intestinal atresia, jejuno-colic anastomosis, low-resource setting, short bowel syndrome

## Abstract

**Introduction:**

Gastroschisis is a congenital abdominal wall defect. The complex form, especially with extensive jejunoileal atresia, is rare and carries high morbidity. Reports of near-total atresia with a preserved colon are scarce, particularly in resource-limited settings.

**Case presentation:**

A 5-day-old male presented with bilious vomiting and failure to pass meconium. Operative findings revealed gastroschisis with an exteriorized, gangrenous cecum and appendix, near-total jejunoileal atresia (only 20 cm of proximal small bowel remaining), malrotation, and a hypoplastic colon. A primary spatulated jejuno-colic anastomosis was performed after resection of non-viable tissue. Enteral feeding began on postoperative day seven.

**Clinical discussion:**

Management of complex gastroschisis with extensive intestinal loss poses major challenges. Staged procedures or proximal diversion, while common, may prolong fluid losses and delay feeding in low-resource contexts. This case highlights that a primary, length-preserving jejuno-colic anastomosis can be a necessary and feasible surgical adaptation to establish immediate enteral continuity when intestinal length is critically limited and staged approaches are untenable.

**Conclusion:**

This case confirms that primary anastomosis can achieve early bowel function. However, the infant later died from short bowel syndrome due to the unavailability of parenteral nutrition. The outcome underscores that, while surgical strategy can be adapted, survival for complex gastroschisis remains dependent on advanced nutritional support, highlighting a critical systemic gap in low-resource neonatal care.

## Introduction

### Background

Gastroschisis is a congenital anterior abdominal wall defect characterized by the herniation of abdominal viscera through a paraumbilical defect, typically to the right of the umbilical cord insertion, without a protective sac^[^1,2^]^. The exposed bowel is directly bathed in amniotic fluid, predisposing it to inflammation, edema, and varying degrees of intestinal dysfunction. The global incidence of gastroschisis has increased over recent decades, with a higher prevalence reported among younger mothers and in certain geographic regions.

HIGHLIGHTS

- This report describes a rare presentation of complex gastroschisis, characterized by near-total jejunoileal atresia and an exteriorized cecum.
- Approximately 20 cm of proximal small bowel remained, necessitating a jejuno-colic anastomosis.
- The resource-limited setting precluded the provision of parenteral nutrition, which contributed to patient mortality.
- The case demonstrates surgical decision-making in scenarios where the bowel length is critically limited.
- This case highlights the necessity for enhanced neonatal nutritional support systems in low- and middle-income countries.

Clinically, gastroschisis is classified into simple and complex forms. Complex gastroschisis is defined by the presence of associated intestinal complications such as atresia, stenosis, perforation, necrosis, or volvulus and is consistently associated with increased morbidity, prolonged hospitalization, delayed enteral feeding, and higher mortality compared to simple gastroschisis^[^3,4^]^. Among these complications, intestinal atresia, particularly jejunoileal atresia, represents a major determinant of outcome and surgical complexity.

Management strategies for gastroschisis focus on early bowel protection, staged or primary abdominal wall closure, and restoration of intestinal continuity when feasible. However, in complex cases, especially those associated with extensive intestinal loss or abnormal anatomy, operative decision-making must balance bowel preservation, functional recovery, and the risk of postoperative complications^[^2,5^]^.

### Rationale

Near-total jejunoileal atresia associated with gastroschisis is rare and represents an extreme form of complex gastroschisis. Published data indicate that infants with gastroschisis complicated by intestinal atresia experience significantly worse outcomes compared with those without atresia, particularly when bowel length is critically reduced^[^3,6^]^. Decisions regarding primary anastomosis, bowel tapering, staged reconstruction, or proximal diversion remain controversial and are often influenced by intraoperative findings, bowel viability, and available postoperative nutritional support.

This case is notable for the presence of near-total jejunoileal atresia with an abnormal jejuno-colic continuity, exteriorized cecum, and gangrenous appendix – an anatomic constellation that is infrequently described in the literature. Reporting this case is important because it highlights operative decision-making in a resource-limited setting, where access to prolonged parenteral nutrition (PN), bowel-lengthening procedures, and multidisciplinary intestinal rehabilitation programs may be limited. The case contributes practical insight into bowel-preserving surgical strategies and their short-term functional outcomes in low- and middle-income countries (LMIC) contexts, where management principles derived from high-income settings may not always be directly applicable^[^2,5^]^. This case report has been prepared in line with the Surgical CAse REport Guideline checklist^[^6^]^.

## Case presentation

A 5-day-old male neonate was referred from a regional hospital with bilious vomiting since birth and failure to pass meconium. He was born at 37 + 2 weeks via spontaneous vaginal delivery, weighing 2900 g. Antenatal care was recorded as regular, but no prenatal imaging was available.

**Figure 1.**
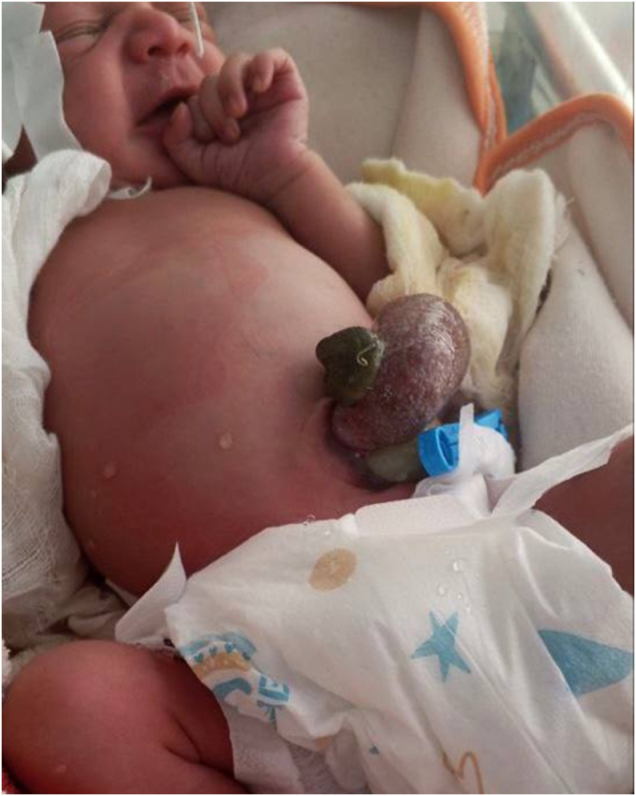
Preoperative photograph demonstrating exteriorized bowel loops to the right of the umbilicus, consistent with gastroschisis.

Upon arrival, the neonate weighed 2655 g. Vital signs were: heart rate 156 per minute, respiratory rate 40 per minute, temperature 36.5°C, and oxygen saturation 98% on room air. The patient was active, without dysmorphic features, but was mildly dehydrated. Urine output was present, but no stool had been passed. The abdomen was mildly distended, and a bowel mass was observed to the right of the umbilicus (Fig. 1), consistent with gastroschisis. Intravenous access was established, fluids were started, and initial laboratory tests were within normal limits.

After stabilization, the infant was taken to the operating room. Nasogastric decompression yielded 20–30 mL of bilious fluid.

### Intraoperative findings

**Figure 2.**
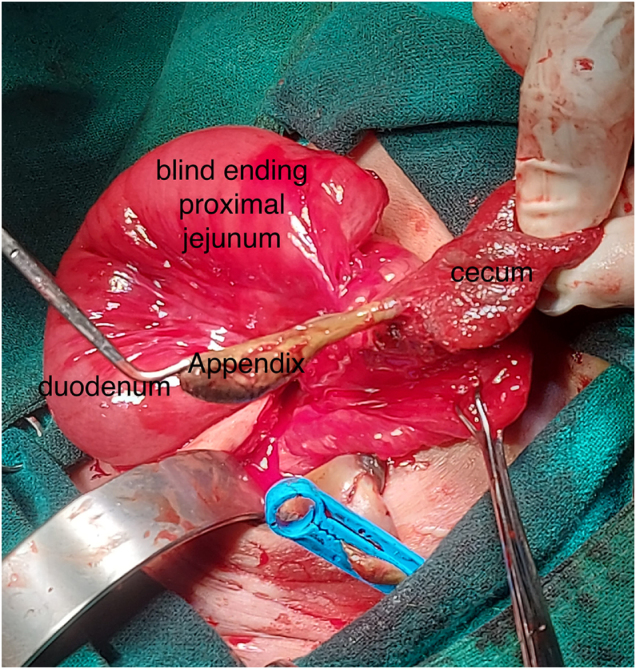
Intraoperative image showing a blind-ending jejunum and a gangrenous appendix.

**Figure 3.**
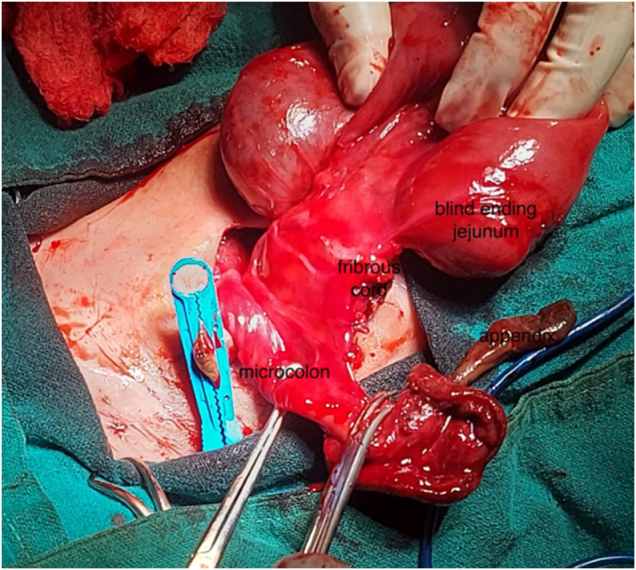
Intraoperative image showing the fibrous cord connecting the jejunum to the colon.

The abdominal wall defect was extended transversely and laterally. Exploration revealed gastroschisis with an exteriorized cecum, its inner surface turned outward, and a gangrenous appendix. Figure 2 illustrates the condition of the appendix and nearby tissues, highlighting the importance of assessing tissue viability during surgery and the decision to resect non-viable segments. Only about 20 cm of proximal small bowel (duodenum and a short part of the jejunum) remained. The ileum and most of the jejunum were missing. A fibrous cord linked the jejunal remnant to a markedly hypoplastic colon about 7 cm long (Fig. 3), which continued to a normal rectum and patent anus. Malrotation was present, but no volvulus was identified.

### Operative management

The gangrenous appendix and the everted cecum were resected. Due to the complete absence of the distal small intestine, a jejuno-colic end-to-end anastomosis was performed. The abdomen was closed without tension, and no silo was required due to the reduced bowel volume.

### Postoperative course

The infant was admitted to the Neonatal Intensive Care Unit. PN could not be administered because neonatal Total Paraentral Nutrition and suitable central lines were unavailable. Nasogastric aspirates ranged from 80 to 200 mL per day during the first week after surgery and then decreased. Enteral feeding was initiated on postoperative day 7 with 2 mL of expressed breast milk every 3–4 hours, gradually increasing as tolerated. The infant began passing stool again.

A subdermal hematoma developed at the incision site. It was managed conservatively after reopening the wound and administering vitamin K. No fascial breakdown occurred.

By postoperative day 14, the infant tolerated increased gavage feeds, although intake remained below age-appropriate norms due to short bowel syndrome (SBS). The patient was transferred to a specialized pediatric nutritional facility in stable condition, tolerating small feeds, and with a healing surgical wound. This facility is primarily dedicated to the management of severe acute malnutrition and focuses on enteral nutritional rehabilitation. It is not a tertiary care center and does not have access to PN, which is unavailable nationwide in our setting. Transfer was, therefore, undertaken only after clinical stabilization in our unit, where the patient was closely monitored for early postoperative complications such as an anastomotic leak and abdominal compartment syndrome.

Despite initial postoperative improvement, the infant developed progressive signs of intestinal failure due to the unavailability of PN and died in the fourth postoperative week.

## Discussion

Complex gastroschisis represents a distinct clinical entity with substantially higher morbidity and mortality compared to simple gastroschisis, primarily driven by associated intestinal pathologies such as atresia, necrosis, or volvulus^[^1,2^]^. Intestinal atresia in this setting is thought to arise from in-utero vascular compromise related to bowel herniation or constriction at the abdominal wall defect^[^3,7^]^. Contemporary studies confirm that complex gastroschisis, which is frequently defined by the presence of intestinal atresia, is associated with a prolonged duration of PN and hospital stay^[^4^]^. The management of gastroschisis complicated by intestinal atresia presents a significant surgical dilemma, balancing bowel preservation, restoration of continuity, and the prevention of complications such as SBS^[^5,8^]^.

In well-resourced settings, a staged approach to atresia repair has been advocated. Fleet and de la Hunt described a protocol of initial abdominal wall closure with electively delayed primary anastomosis at 4–6 weeks, reporting good outcomes^[^9^]^. This strategy aims to allow reduction of bowel edema and inflammation, facilitating a safer anastomosis at a later date. Similarly, the use of a proximal diverting stoma remains a common initial step, particularly when bowel viability or size discrepancy is a concern^[^5^]^. However, these approaches inherently depend on the ability to provide sustained, complication-free PN and to manage potential high-output enterostomy losses for an extended period.

Confronted with the severe anatomical challenge of near-total jejunoileal atresia in a setting where neonatal PN was unavailable, our decision-making pivoted from standard protocols. The imperative shifted from mitigating anastomotic risk to achieving immediate enteral continuity. This was the only viable pathway to eventual enteral autonomy. Intraoperatively, the presence of viable, albeit limited, proximal jejunum and colon permitted a primary anastomosis. We performed a spatulated jejuno-colic anastomosis without proximal tapering or diversion, a decision guided by the principle of maximal bowel length preservation^[^3,5^]^ and the option for primary repair when bowel condition allows^[^8^]^. Avoiding a stoma was a deliberate choice to eliminate the catastrophic risk of dehydration and electrolyte depletion from high-output losses in a PN-void environment.

The postoperative course demonstrated the functional success of this adapted strategy. Bowel activity returned, and enteral feeding was initiated, confirming the patency and function of the anastomosis. This highlights that a single-stage, length-preserving reconstruction can achieve early gastrointestinal continuity. However, the infant’s subsequent deterioration and death from complications of SBS during the fourth postoperative week illustrate the profound physiological limits of surgical technique alone. With approximately 20 cm of residual small bowel, the infant had severe intestinal failure, a condition survivable only with sustained advanced nutritional support^[^8^]^. The absence of PN was therefore not a contributing factor but the determining factor in the outcome. This starkly underscores that in the management of extreme short bowel, surgical strategy and postoperative nutritional infrastructure are inextricably linked; success in one domain cannot compensate for failure in the other.

In conclusion, this case of near-total jejunoileal atresia with gastroschisis demonstrates that, in resource-limited environments, surgical principles must be adapted to systemic constraints. A primary bowel-preserving anastomosis without diversion can be a necessary and feasible option to establish immediate enteral continuity when staged approaches are untenable. However, the outcome definitively highlights that the prognosis for infants with complex gastroschisis and severe SBS remains inextricably dependent on access to multidisciplinary intestinal rehabilitation programs, with PN as a cornerstone therapy^[^4,8^]^. This report serves as a compelling call to address the critical gap in postoperative nutritional support systems to improve survival for neonates with complex surgical conditions in LMIC.

## Conclusion

In conclusion, while this case shows that surgical technique can be adapted to achieve gastrointestinal continuity in the most severe forms of complex gastroschisis, it delivers a stark reminder that physiology imposes an absolute limit. The infant’s death from complications of SBS, solely due to the unavailability of PN, transforms this report from a surgical lesson into a compelling systemic critique. To improve outcomes, efforts to advance neonatal surgery in LMIC must be matched by equally vigorous initiatives to build the nutritional support systems that make such surgery survivable.

## Data Availability

Not applicable.

## Patient perspective

The infant’s parents were counseled extensively on the diagnosis, the planned surgical approach, and the critical postoperative challenges, including the high risk of mortality due to the unavailability of life-sustaining PN. They provided consent for all procedures and for this publication.
